# Immunohistochemical Analysis of Retinoblastoma and β-Catenin as an Assistant Tool in the Differential Diagnosis between Crohn's Disease and Ulcerative Colitis

**DOI:** 10.1371/journal.pone.0070786

**Published:** 2013-08-14

**Authors:** Rossana Colla Soletti, Nathassya Accioly Lins Vidal Rodrigues, Deborah Biasoli, Ronir Raggio Luiz, Heitor Siffert Pereira de Souza, Helena Lobo Borges

**Affiliations:** 1 Instituto de Ciências Biomédicas, Universidade Federal do Rio de Janeiro, Rio de Janeiro, RJ, Brazil; 2 Instituto de Estudos de Saúde Coletiva (IESC), Universidade Federal do Rio de Janeiro, Rio de Janeiro, RJ, Brazil; 3 Departamento de Clínica Médica, Hospital Universitário Clementino Fraga Filho, Universidade Federal do Rio de Janeiro, Rio de Janeiro, RJ, Brazil; The Chinese University of Hong Kong, Hong Kong

## Abstract

In about 10–15% of patients with inflammatory bowel diseases (IBD) there is no clear definitive differential diagnosis between Crohn's disease (CD) and ulcerative colitis (UC) and the disease is classified as indeterminate colitis. Since pharmacological and surgical treatments differ in CD and UC, establishing a correct diagnosis is critical. The aim of this work was to access the expression profile of proteins involved in colonic inflammation and cancer in samples from CD and UC. For that, colon samples from 24 CD, 21 UC and 10 control patients were processed for immunohistochemistry using anti-phosphorylated RB at Ser^807/811^ and anti-β-catenin. Crypts were blinded, analyzed and counted for phosphorylated RB-positive (phospho-RB) cells or scored for positive β-catenin staining. Western blot was used for confirming immuhistochemical results: RB phosphorylation was significantly greater in colon samples from patients with CD compared with UC (p<0.005). In contrast, the expression of β-catenin was significantly increased in UC compared with CD (p<0.005) samples. Phospho-RB and β-catenin are negatively correlated (CC: −0.573; p = 0.001). A positive phospho-RB test yielded high levels of sensitivity, specificity, negative and positive predictive values, and accuracy for the diagnosis of CD against UC. This work indicates that RB phosphorylation and β-catenin nuclear translocation are differently expressed in CD and UC, and provide novel insights into the pathogenic mechanisms of IBD. In particular, rates of phospho-RB-positive cells in mucosal samples emerge as a promising tool for the differential diagnosis of patients with IBD.

## Introduction

Crohn's disease (CD) and ulcerative colitis (UC) are the two main forms of inflammatory bowel diseases (IBD), characterized by intestinal inflammation and ulceration of unknown etiology [1], [2]. Although CD and UC share similar pathophysiological mechanisms, such as immune activation, leukocyte infiltration and increased colonic vascular density, they usually present important differences regarding anatomical localization, histopathological findings, disease progression and therapeutic response [3], [4]. The diagnosis of CD and UC currently relies on a combination of clinical, endoscopic, histological, and imaging parameters [5], [6]. Nevertheless, a subset of patients remains indeterminate in their diagnosis, whenever presenting exclusively with colitis [7], [8].

In fact, the differentiation between Crohn's colitis and UC can be challenging sometimes, even to experienced clinicians, and the rates of indeterminate colitis, also known as IBD-unclassified (IBD-U) has not changed significantly over the past thirty years [9], [10]. Of note, since pharmacological and particularly surgical treatments, as well as the concerns of associated tumorigenesis differ in CD and UC [11], the establishment of a correct diagnosis is of paramount importance, and critically influences the disease outcome.

Over the last decades, advances in molecular biology techniques provided a better understanding of the pathogenic mechanisms underlying IBD. To elucidate the molecular events involved in IBD pathogenesis, the efforts of some research groups have been focused on the analysis of protein expression and the investigation of susceptibility genes [12]. Lawrance and co-workers [13] showed significant differences in the expression profile of 170 genes in CD and UC. Christophi and collaborators [14] also showed that several inflammatory mediators, oxidative stress inducers, proteases and mucosal genes were differently regulated in CD and UC, suggesting that each of these diseases have different molecular interactions. However, to this date, no study addressed the differential expression of β-catenin and retinoblastoma protein (RB), two key regulators of colonic proliferation, inflammation, and tumorigenesis, in CD and UC.

β-catenin is manly detected as part of the adherent junction component, decorating the basolateral membrane of epithelial cells. In the bottom of colonic crypts, however, progenitor cells accumulate cytoplasmic/nuclear β-catenin that binds to members of the transcriptional factors family lymphoid enhancer factor/T-cell factor (LEF/TCF) to drive proliferation [15]. It was already observed that dysplastic areas of UC surgical specimens demonstrated a strong and diffuse nucleocytoplasmic β-catenin immunolabeling [16]. However, β-catenin expression and localization in CD surgical samples has not been investigated, so far. Previous findings from Sturm and co-workers [17] showed that T cells isolated from the intestinal mucosa of CD patients express higher phosphorylation levels of RB than UC T cells. The involvement of RB in colonic inflammation has also been investigated. A mutation in Rb caspase cleavage site (*Rb-MI*) protects mice colonic epithelium from LPS-induced cell death [18] and from dextran sodium sulfate (DSS)-induced cell death and ulceration [19], [20]. These data suggest that RB could protect intestinal epithelial cells against tumor necrosis factor (TNF)-induced cell death in human pathology. Since TNF-α production is increased in IBD [21], we decided to investigate RB expression and phosphorylation levels in epithelial cells from CD and UC patients.

In this work, we show that there is a remarkable difference in the expression of β-catenin and RB phosphorylation (phospho-RB). The colonic epithelium from UC patients shows an increase in β-catenin cytoplasmic and nuclear accumulation, whereas epithelial cells from CD patients present an increase in RB phosphorylation.

## Materials and Methods

### Patients

Forty-seven patients with IBD, 24 with Crohn's disease (CD), 21 with ulcerative colitis (UC), and 2 with IBD unclassified (IBDU) were enrolled in this study. The diagnosis of IBD was confirmed by the routine clinical, radiological, endoscopic and histological criteria.

Patients with CD consisted of 13 women and 11 men, with a mean age of 38 years (range 18–65 years). All patients had active CD at the time of the study, based on the Simple Index of Harvey and Bradshaw [22]. At the time of the study, patients had ileocolonic or colonic disease that had been present from one to 14 years (mean duration: 7.3 years). In regard to treatment, four patients were on corticosteroids only, four on corticosteroids and aminosalicylates, four on corticosteroids and antibiotics, 10 on immunosupressors and aminosalicylates, and two received infliximab and immunosupressors.

Patients with UC comprised 13 women and eight men, with a mean age of 41 years (range: 23–70 years). The mean duration of UC was 8.5 years (range: 1–22 years), at the time of the study. According to Truelove and Witts [23], 13 patients had moderate disease activity, and eight had severe disease at the time of the study. Ten patients were on aminosalicylates only, five on corticosteroids and aminosalicylates, six on immunosupressors and aminosalicylates. Twelve patients had limited left-sided colitis and nine had pancolitis.

Of the two cases of IBDU, both presented moderate to severe pancolonic disease, where criteria for either CD or UC could not be definitively established by the time of the sample collection for the study. One of them was a 28-year old woman with a history of undiagnosed hemorrhagic diarrhea, with abdominal pain, weight loss, and anemia, which had been present for the last 3 years. The other patient was a 50-year old woman with a 4-year history of undiagnosed diarrhea, with abdominal pain and tenesmus, peripheral arthritis and scars on the lower limbs, possibly compatible with previous pyoderma gangrenosum. At the time of colonoscopy and biopsies, both were taking immunossupressors and aminosalicylates.

Control patients consisted of five individuals with diverticular disease, four with benign polyps, and one with benign intestinal obstruction. The control group comprised five men and five women, with a mean age of 44 years (range: 18–64 years). None of the controls were taking any medication by the time of the study. The study protocol was approved by the Ethical Committee of the University Hospital, Federal University of Rio de Janeiro, and written informed consent was obtained from all patients (approval number # 188/08).

### Colonic specimens

Biopsy specimens from individuals undergoing diagnostic or surveillance colonoscopy, or surgical resections were obtained at the *Hospital Universitário Clementino Fraga Filho*, Rio de Janeiro.

Histologically normal tissue was obtained from at least 10 cm from the polyps or diverticula. IBD samples were all inflamed colonic specimens from patients with CD and UC. All control patients had normal colonoscopy, and the mucosal biopsies were histologically normal. Specimens were fixed in 40 g/l formaldehyde saline, embedded in paraffin, cut into serial sections of 5 μm, and submitted to the different staining procedures.

### Immunohistochemical evaluation

Immunohistochemical staining was performed using a LSAB+ System-HRP (DakoCytomation, Denmark) kit according to the manufacturer's instructions. Antigen retrieval was performed boiling the slides in 10 mM citrate buffer pH 6.0 for 10 min. Sections were incubated with anti-phosphorylated RB at Ser^807/811^ (1∶100, Cell Signaling Technology, Danvers, MA) and anti-β-catenin (1∶100, BD Biosciences, San Jose, CA). Slides were developed using 3,3′-diaminobenzidine tetrahydrochloride and counterstained with methyl green (Sigma Chemical Co, St Louis, USA).

For the imunofluorescence studies, slides were incubated with anti-phosphorylated RB at Ser^807/811^ (1∶100, Cell Signaling Technology, Danvers, MA), at Thr^821/826^ (1∶50, Santa Cruz Biotechnology, Santa Cruz, CA) and anti-β-catenin (1∶100, BD Biosciences, San Jose, CA). The secondary antibodies used were donkey anti-rabbit conjugated to Alexa Fluor 488 (green) or goat anti-mouse (Invitrogen, Carlsbad, CA) conjugated to Alexa Fluor 594 (red). Nuclei were stained with DAPI (4′-6-diamidino-2-phenylindole). Fluorescence intensity was evaluated using Photoshop 7.0.1 software (Adobe), by analyzing at least five representative fields for each sample obtained through a fluorescence microscope (Nikon TE 300) under 400x magnification.

### Quantitative Assessment of Colon Sections

Two experienced observers, who were unaware of the experimental data, carried out all histomorphological analyses of tissue sections. Any epithelial cells exhibiting identifiable reactivity distinct from background were regarded as positive. For counting phosphorylated RB-positive epithelial cells, sections of at least 10 crypts from each colon sample were analyzed, and percentages were defined by the number of immunoreactive cells in relation to total cells (immunoreactive and non-immunoreactive cells). Results are presented as the mean ± standard error of the mean (S.E.M).

For analyzing the expression of β-catenin, at least three representative photomicrographs (200× magnification) of each case were obtained. A semi-quantitative analysis was then performed, based on the staining intensity (at nuclei, cytoplasm and cell membrane): 0 (no staining), 1 (weak staining), 2 (medium staining) and 3 (strong staining). Representative photomicrographs of this score are shown in Fig. 3B. For the purpose of comparing results among groups, staining intensity categories were converted into numerical and the average reading was recorded.

### Protein extraction from paraffin-embedded tissues

Protein extraction from paraffin-embedded colonic biopsies was carried out according to Nirmalan et al [24]. For each sample, three 10 µm sections from CD, UC and control patients were used. Samples were collected into a glass tube, deparaffinized in three baths of xylene (10 min each), rehydrated in 100%, 90%, 80% and 70% ethanol baths, centrifuged and minced with scissors for 15 s in PBS (phosphate-buffered saline) buffer on an ice-cold plate. Then, each sample was centrifuged, ressuspended in 150 µl freshly prepared Laemmli buffer (100 mM Tris-HCl pH 6.8, 2% w/v SDS, 20% v/v glycerol, 4% v/v β-mercaptoethanol), heated at 105°C for 20 min, and cooled for 5 min on ice. Protein concentration was calculated using the Bradford assay [25].

### Western blot

Similar amounts (20 μg) of total protein were loaded on SDS-PAGE, transferred onto a PVDF membrane and probed with primary antibodies overnight at 4°C. HRP conjugated secondary antibodies were incubated for 2 h at room temperature. Primary antibodies used were anti-phosphorylated RB at Ser^807/811^ (Cell Signaling Technology, Danvers, USA), anti-β-catenin (BD Biosciences, San Jose, USA) and anti alpha-Tubulin (Sigma Aldrich, St Louis, USA). Secondary antibodies used were Horseradish peroxidase (HRP)-conjugated secondary antibodies purchased from Invitrogen, CA. In the quantification process, we used alpha-tubulin densitometry of each sample as a normalizing factor for the quantification of phospho-RB and β-catenin.

### Statistical analysis

Statistical analysis was performed using the statistical software SPSS for Windows (Version 10.0.1, SPSS Inc., 1989–1999, USA). Statistical differences among the experimental groups were determined by ANOVA test in which pair wise multiple comparisons were carried out using the Dunnett's test and Tukey's multiple comparisons test. Correlation between phospho-RB and β-catenin was assessed using the Spearman's rank correlation coefficient. Relative operating characteristic curves (ROC) were applied to tests in order to define thresholds. The ability to discriminate between CD and UC was determined in terms of sensibility, specificity, positive predictive values, negative predictive values, and accuracy based on the clinical, endoscopic, histological, and radiologic parameters as the gold standard for diagnosis. The level of significance was set at *P*<0.05.

## Results

### Retinoblastoma phosphorylation is increased in CD colonic samples

**Figure 1:**
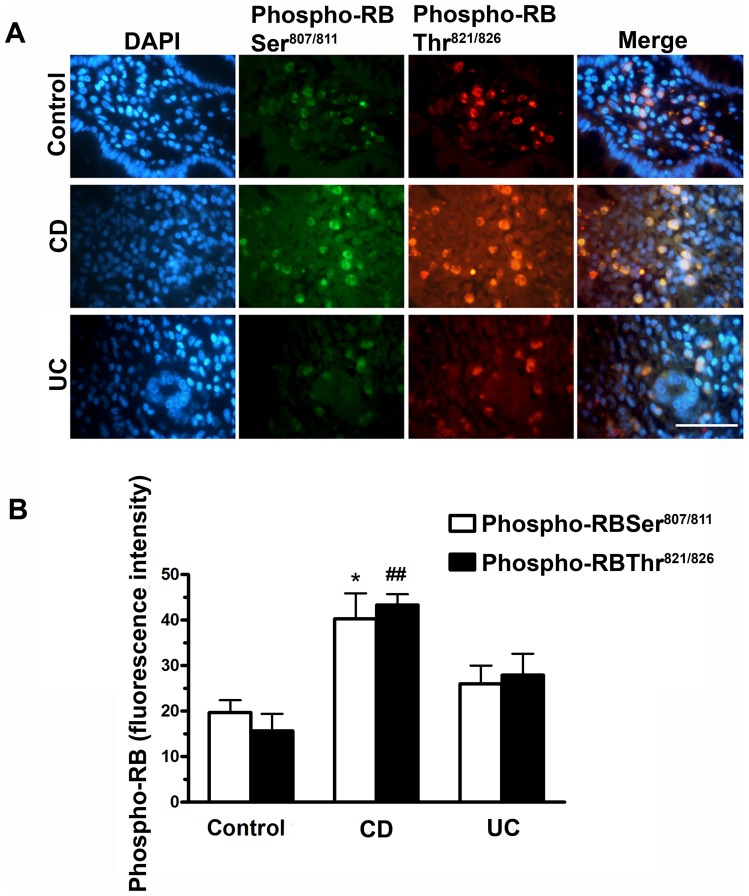
Immunofluorescence staining for phosphorylated RB is increased in colon biopsies from CD patients. A. RB phosphorylation was detected in colon biopsies from control (non inflamed; n = 5), CD (n = 7) and UC patients (n = 6) by immunofluorescence staining using phosphorylated RB Ser^807/811^ and phosphorylated RB Thr^821/826^ antibodies. Nuclei were stained with 4′,6-diamidino-2-phenylindole (DAPI). Scale bar = 50 µm. B. Histogram showing RB fluorescence staining intensity in Ser^807/811^ (white bars) and Thr^821/826^ (black bars) residues. *p<0.05 when compared with percentage of phosphorylated RB Ser^807/811^-positive cells in UC samples and ^##^p<0.01 when compared with percentage of phosphorylated RB Thr^821/826^ -positive cells in UC samples.

Previous studies from our group showed that apoptosis and ulceration induced by colitis in mice depends on Rb cleavage by caspase [18], [19], supporting a significant role for RB in intestinal inflammation. Therefore, we sought to determine the potential involvement of retinoblastoma phosphorylation in IBD by analyzing colonic samples from patients. Colonic specimens from 7 patients with CD and 6 patients with UC were firstly screened by immunofluorescence using antibodies directed against serine (anti-phospho RB Ser^807/811^) and threonine (anti-phospho RB Thr^821/836^) residues. Fig. 1A shows that RB phosphorylation is increased in colonic samples from patients with CD but not with UC. When compared with control colonic samples, a significantly different phosphorylation of RB Ser^807/811^ (p<0.05) and Thr^821/836^ (p<0.01) was observed (Fig. 1B). The levels of RB phosphorylation staining in UC and control samples were not significantly different, although there was a significant difference between RB phosphorylation of CD and UC samples.

**Figure 2:**
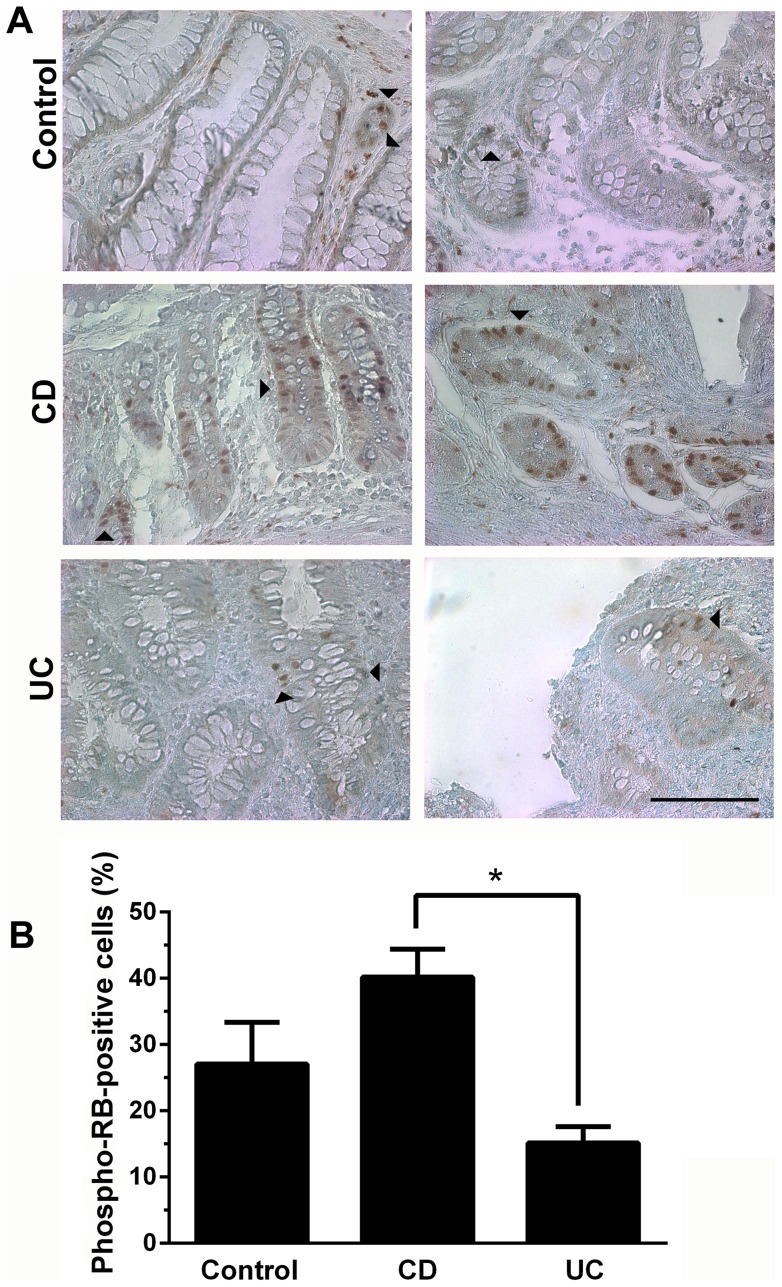
RB phosphorylation is increased in colonic epithelial cells from CD patients. A. RB phosphorylation was detected in colon biopsies from control (non inflamed), CD and UC patients by immunohistochemistry using phosphorylated RB Ser^807/811^antibody. Slides were counterstained with methyl green. Scale bar = 50 µm. Arrowheads point to phosphorylated RB-positive cells. B. Histogram shows the percentage of phosphorylated RB-positive cells in control (n = 5), CD (n = 15) and UC (n = 13) samples. *p<0.0001 when compared with phospho-RB Ser^807/811^ staining in control samples. Scale bar = 50 µm.

To evaluate the RB phosphorylation profile within the colonic samples we performed immunohistochemistry analysis in 15 specimens of CD patients and 13 from UC patients. Fig. 2 shows that, in agreement with our previous findings, RB phosphorylation is significantly increased in colonic samples from patients with CD, compared with UC patients. Of the two patients with IBDU pancolitis, the expression of phosphorylated RB was high (67%) in the 28-year old woman, and relatively low (26%) in the 50-year old woman intestinal specimens.

### β-catenin expression is increased in UC colonic samples

**Figure 3:**
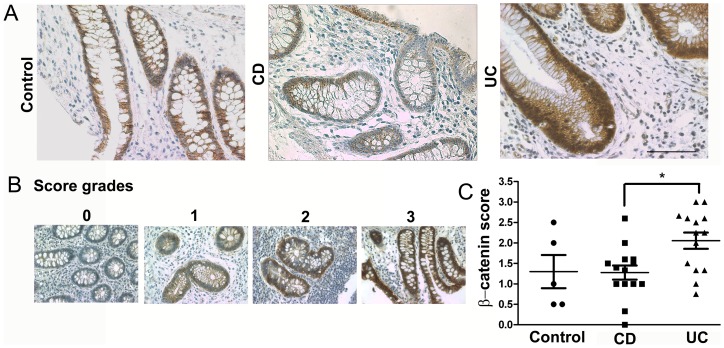
β-catenin is overexpressed in colonic samples from UC patients. A. Colonic biopsies from control (non inflamed), CD and UC patients were stained by immunohistochemistry using anti-β-catenin antibody. Slides were counterstained with methyl green. Scale bar = 50 µm. B. Representative pictures of β-catenin staining scores (0 to 3). C. Graph shows the mean scores of β-catenin expression intensity in control (n = 5), CD (n = 14) and UC (n = 14) samples. *p = 0.019 when compared with β-catenin expression scores in CD samples.

The expression of β-catenin was analyzed in regard to subcellular localization (cytoplasm, nuclei and cell membranes), and staining intensity, using a semi-quantitative score. β-catenin expression pattern and subcellular localization in colonic samples from CD patients is similar to that found in control non-inflamed colon (Fig. 3A). Interestingly, colon samples from UC patients present an accumulation of β-catenin in cytoplasmic and nuclear compartments. The staining intensity of β-catenin was almost two times higher in UC than in CD samples (Fig. 3C). Among all UC patients, even the two who scored below 1 for β-catenin staining intensity also had a small percentage of RB phosphorylation.

In regard to IBDU patients, in contrast to the results observed with phosphorylated RB, the staining intensity of β-catenin was relatively low (score 1) in the 28-year old woman, and high (score 3) in the 50-year old woman intestinal specimens.

**Figure 4:**
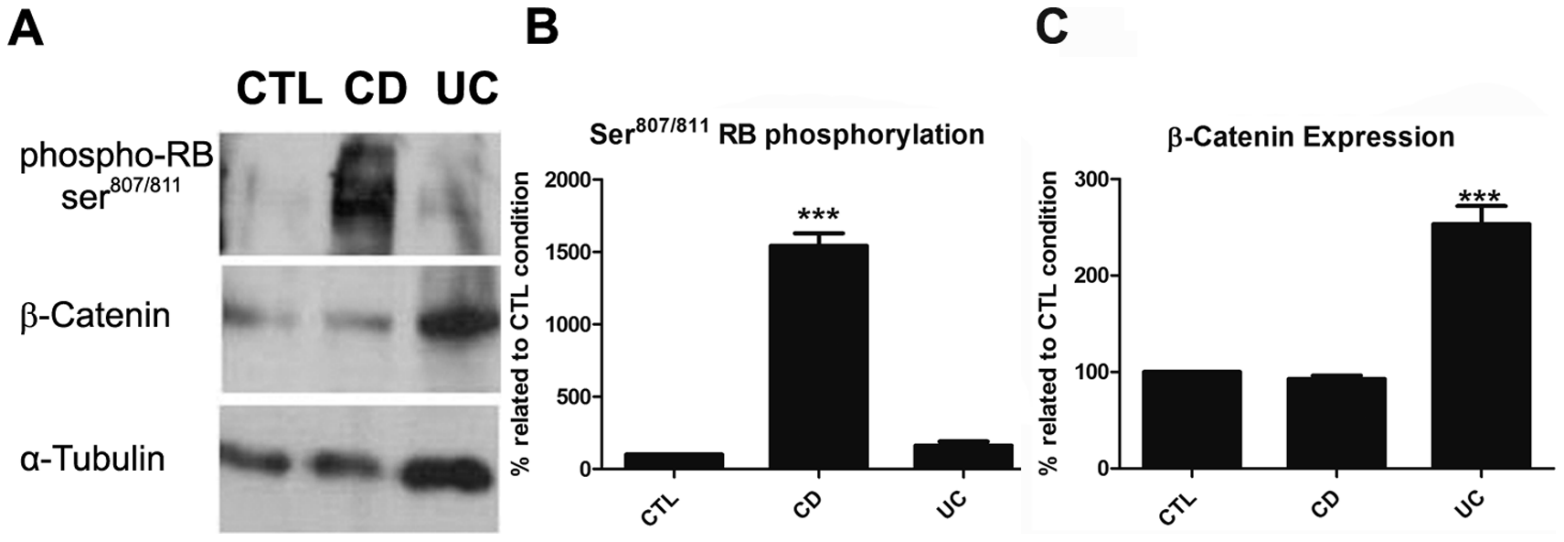
Immunoblotting detection of phospho-Rb and β-catenin in samples from control, CD and UC samples. A. Representative images of imunnoblots from control (CTL, n = 4), CD (n = 4) and UC (n = 4) samples, detecting β-catenin expression and RB phosphorylation at ser 807/811 residues (phospho-RB ser^807/811^). α-tubulin was used as loading control. B. Histogram showing the quantification of RB phosphorylation at Ser^807/811^ residues in control (CTL), CD and UC samples by band densitometry. C. Histogram showing the quantification of β-catenin expression in control (CTL), CD and UC samples by band densitometry. ***p = 0.005 when compared with UC.

To confirm these data, western blot analysis were performed with protein samples extracted from paraffin-embedded colonic biopsies (4 CD, 4 UC and 4 control samples). The RB phosphorylation and β-catenin expression were measured in parallel using the same patient sample from each condition (CD, UC and control). According to Fig. 4, CD samples showed eight times more RB phosphorylation than UC samples. On the other hand, UC samples showed a 2.5 times increase in β-catenin expression than CD samples, validating the results previously obtained by imunohistochemisty.

No significant association was detected between any of the immunohistochemical results and the demographic data, clinical characteristics, or the different types of treatment of the patients with IBD.

**Figure 5:**
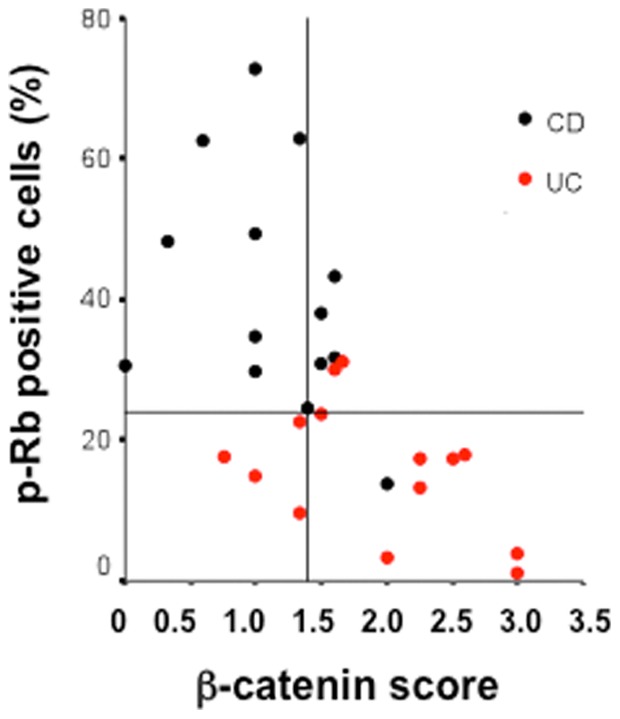
Scatter-plot showing the correlation between phospho-RB and b-catenin among patients with CD and UC. ROC curves determined thresholds for establishing positive test values. Phospho-RB is expressed as percentages, while β-catenin is expressed as intensity scores.

Analysis of data distribution in a scatter plot (Fig. 5) clearly shows a negative correlation between phospho-RB and β-catenin in the groups. The Spearman's rank correlation coefficient, calculated between phospho-RB and β-catenin, was r = −0.573; p = 0.001. After running ROC curves for each variable, cut-off points were defined in order to classify patients according to the diagnosis of either CD or UC. A positive diagnosis of CD was considered when phospho-RB positive cells ≥24%, and when β-catenin score ≤1.4 (Fig. 5). Results obtained were used to assess the diagnostic capabilities of each test. Among the tests, the percentage of phospho-RB positive cells showed remarkable indexes, from sensitivity to accuracy, and to our knowledge, with unparalleled results in the literature (Table 1).

**Table 1:** Diagnostic capability of phospho-RB and β-catenin immunhistochemical tests in differentiating Crohn's disease from ulcerative colitis.

*Test*	*Sensitivity*	*Specificity*	*PPV*	*NPV*		*Accuracy*
Phospho-RB (+)	93.3	86.7	87.5	92.9	90.0	
β-catenin (−)	57.1	71.4	66.7	62.5	64.3	

All values are presented as percentages. Cut-off values were determined by ROC curves, establishing as positive tests: phospho-RB-positive cells ≥24%; and β-catenin score ≤1.4. The positive endpoint was arbitrarily considered for the diagnosis of Crohn's disease. NPV, negative predictive value; PPV, positive predictive value.

**Figure 6:**
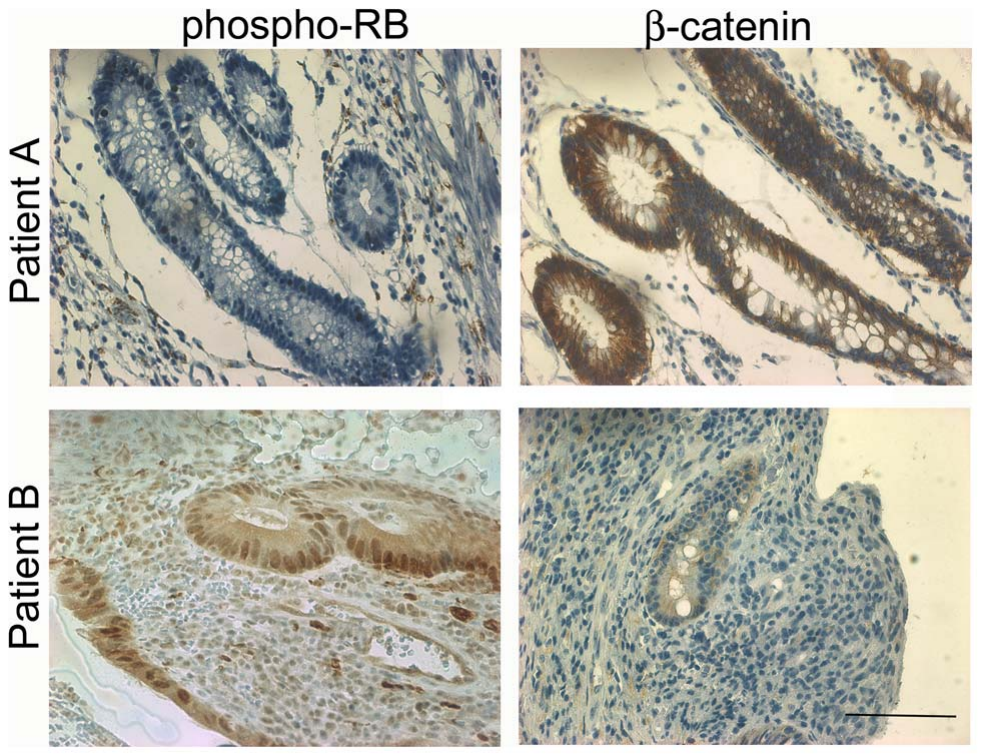
Phospho-RB and β-catenin staining pattern helps the differential diagnose of IBDU cases. A. Low phospho-RB ser^807/811^and high β-catenin staining in a 50-year old woman patient lately diagnosed with UC (Patient A). B. High phospho-RB ser^807/811^and low β-catenin staining in a 28-year old woman lately diagnosed with CD (Patient B). Slides were counterstained with methyl green. Scale bar = 50 µm.

Of note, after all samples have been collected and the experiments have been performed and analyzed, the two cases of indeterminate colitis developed additional unequivocal characteristics that allowed the establishment of a specific diagnosis during the two-year follow-up period. Interestingly, the 50-year old woman with low phospho-RB and high β-catenin expression lately developed cardinal features of UC, presenting a more distal disease with extensive ulceration, with crypt microabscesses. On the other hand, the 28-year old woman with high phospho-RB and low β-catenin expression developed a clear typical CD, with ileitis, penetrating lesions in the colon, and perianal disease. Because of that, the 2 IBDU samples were now classified as CD or UC and the RB phosphorylation and β-catenin staining pattern were compatible with the diseases (Fig. 6).

## Discussion

Crohn's disease (CD) and ulcerative colitis (UC) are complex and polygenic diseases caused by deregulation in genes that contribute to innate and adaptive immune responses in predisposed individuals. Despite similar clinical and demographic characteristics, CD and UC carry substantial differences in histopathological appearance and disease course, suggesting distinct etiopathogenic processes [8]. Recent studies point out significant differences in the genetic expression profile of CD and UC, corroborating the idea that they constitute distinct molecular entities [13], [14], [26]–[28].

Several proteins have been shown to be implicated in the control of histopathological alterations of CD or UC. We used colon samples of CD and UC patients to verify by immunohistochemistry and by western blot the expression pattern of proteins involved in colonic inflammation and cancer. In our findings, the expression of β-catenin in UC samples was two times higher than in CD samples. On the other hand, phosphorylation of retinoblastoma protein (RB) was markedly increased in CD, but not in UC samples. Determining the expression status of these molecules can greatly contribute in the discrimination between CD and UC, and constitute novel biomarkers potentially influencing the therapeutic management and the clinical outcomes of patients with IBD.

RB was the first tumor suppressor identified [29] and has a critical role in controlling cell cycle. Hypophosphorylated RB controls cell cycle by sequestering the E2F family of transcription factors. Mitogenic signals induce RB hyperphosphorylation through cyclin D/CDK4-6 and cyclin E/CDK2, with consequently E2F release and transcription of growth-associated genes [30]. About 70% of all human cancers have mutations in RB upstream proteins, resulting in RB hyperphosphorylation [31], [32]. RB phosphorylation is also increased in colon tumor cells following inflammatory stimuli [33], [34]. In agreement with our findings, Sturm et al. [17] observed that colonic T cells obtained from CD patients show increased RB phosphorylation than UC-derived T cells. Moreover, DSS-induced colitis increased RB phosphorylation and inactivation in the colonic mucosa [35], [36], resulting in increased proliferation through the E2F1 pathway. This RB hyperphosphorylation is mediated by nitric oxide and is dependent of MEK/ERK (mitogen activated protein kinase/extracellular signal-regulated kinase kinase and PI3K (phosphatidylinositol 3-kinase)/AKT pathways [36].

The high rates of phospho-RB found in mucosal samples of CD patients appear to open a conceptual new approach for understanding pathogenic mechanisms of intestinal diseases. In particular, in addition to the new mechanistic observation, the expression of intestinal phospho-RB also potentially emerges as an invaluable tool for the differentiation between CD and UC. Currently, even after all advances in the field of molecular biology, a thorough biologic marker for differentiating CD colitis from UC is still pending. Traditionally, the combination of a positive anti-*Saccharomyces cerevisiae* antibodies (*ASCA*) test with a negative perinuclear antineutrophil cytoplasmic antibodies (*pANCA*) test has been utilized as serological markers for discriminating cases of CD colitis from UC [37], [38]. However, there are concerns regarding the utility of these tests, because data show that almost half of the patients may not develop *ASCA* or *pANCA* antibodies [39]–[41]. In fact, many studies have shown that patients with diseases other than CD, for example, Behcet disease, ankylosing spondylitis and cystic fibrosis, may also have a higher frequency of ASCA seropositivity than the general population [42]–[44]. In the current study, we present for the first time the analysis of mucosal phospho-RB expression as a potential new surrogate marker for CD, with diagnostic capabilities, in conjunction, better than the ones obtained with combined ASCA and p-ANCA.

The involvement of wnt pathway is a hallmark of colon cancer. Loss or mutation in the tumor suppressor gene *APC* (which controls β-catenin degradation) causes adenomatous transformation of intestinal epithelium [45], [46] and it is regarded as the main cause of colon cancer in humans [47]. Therefore, most epithelial cells in human colorectal cancer (CRC) samples present nuclear or cytoplasmic accumulation of β-catenin [48]. We have shown that nuclear β-catenin translocation protects colon cancer cells from TNF-induced cell death [49], which may have an important role in chronic intestinal inflammation. Lee and co-workers [50] showed that β-catenin nuclear/cytoplasmic translocation occurs not only in sporadic CRC, but also in human colonic biopsies of UC-induced cancer, UC-induced dysplasia and UC. Van Dekken and collaborators [16] also demonstrated a strong and diffuse nucleocytoplasmic β-catenin immunolabeling in dysplastic areas of UC surgical specimens, corroborating our results. Brown et al. [51] showed that mesalamine, a mainstay therapeutic agent for UC, decreases the expression of Akt-phosphorylated β-catenin on intestinal crypts of UC patients and in an animal model of intestinal chronic inflammation (IL-10^−/−^).

Taking into consideration the cases of IBDU enrolled in this study, it is interesting to notice that the disease definition as either CD or UC could almost be anticipated by the findings on the intestinal expression of phospho-RB and β-catenin. Whether or not phospho-RB and β-catenin expression could be utilized as adjunct tools for helping in the differential diagnosis is yet to be determined. Nevertheless, the results on the differential expression of phospho-RB and β-catenin lend additional support to the potential role of those proteins in the pathogenesis of the different types of IBD.

Despite all the new studies showing distinct molecular mechanisms in IBD and differences in protein expressions on CD and UC colonic samples [13], [14], there are no immunohistochemistry-based methods routinely used for IBD diagnosis. Although more research with a greater cohort is needed before immunohistochemical detection of phospho-RB and β-catenin could be routinely applicable as a laboratory test, our findings raise the possibility of using these proteins as potential molecular markers to help in the differentiation between CD and UC, and for elucidating unclassified-IBD cases.

## References

[1] Kirsner JB (2006) The Historical Basis of the Idiopathic Inflammatory Bowel Diseases. Inflamm Bowel Dis 1: 2–26.

[2] Lakatos PL (2006) Recent trends in the epidemiology of inflammatory bowel diseases: Up or down? World J Gastroenterol 2: 6102–6108. 10.3748/wjg.v12.i38.6102 PMC4088101 17036379

[3] van Assche G, Vermeire S, Rutgeerts P (2010) Mucosal healing and anti TNFs in IBD. Curr Drug Targets 11(2): 227–233. 20210770 10.2174/138945010790309902

[4] Bouma G, Strober W (2003) The immunological and genetics basis of inflammatory bowel disease. Nature Rev 3: 521–533. 10.1038/nri1132 12876555

[5] Cohen RD (2003) Inflammatory Bowel Diseases. Humana Press, Totowa.

[6] Kirsner JB (1988) Historical Aspects of Inflammatory Bowel Disease. J Clin Gastroenterol 10: 286–297. 2980764 10.1097/00004836-198806000-00012

[7] Blumberg RS (2008) Crohn Disease. JAMA300: 439–440. 10.1001/jama.300.4.439 18647989

[8] Nikolaus S, Schreiber S (2007) Diagnostics of inflammatory bowel disease. Gastroenterology 133(5): 1670–1689. 17983810 10.1053/j.gastro.2007.09.001

[9] Telakis E, Tsironi E (2008) Indeterminate colitis – definition, diagnosis, characteristics and management. Ann Gastroenterol 21(3): 173–179.

[10] Iskandar HN, Ciorba MA (2012) Biomarkers in inflammatory bowel disease: current practices and recent advances. Transl Res 159(4): 313–325. 22424434 10.1016/j.trsl.2012.01.001 PMC3308116

[11] Van Assche G, Vermeire S, Rutgeerts P (2005) Medical treatment of inflammatory bowel diseases. Curr Opin Gastroenterol 21(4): 443–447. 15930985

[12] Abraham C, Cho JH (2009) Inflammatory bowel disease. N Engl J Med 361(21): 2066–2078. 19923578 10.1056/NEJMra0804647 PMC3491806

[13] Lawrance IC, Fiocchi C, Chakravarti S (2001) Ulcerative colitis and Crohn's disease: distinctive gene expression profiles and novel susceptibility candidate genes. Hum Mol Genet 10(5): 445–456. 11181568 10.1093/hmg/10.5.445

[14] Christophi GP, Rong R, Holtzapple PG, Massa PT, Landas SK (2012) Immune markers and differential signaling networks in ulcerative colitis and Crohn's disease. Inflamm Bowel Dis 29. 10.1002/ibd.22957 PMC3407828 22467146

[15] Van de Wetering M, Sancho E, Verweij C, de Lau W, Oving I, et al (2002) The beta-catenin/TCF-4 complex imposes a crypt progenitor phenotype on colorectal cancer cells. Cell 111: 241–250. 12408868 10.1016/s0092-8674(02)01014-0

[16] Van Dekken H, Wink JC, Vissers KJ, Franken PF, Ruud Schouten W, et al (2007) Wnt pathway-related gene expression during malignant progression in ulcerative colitis. Acta Histochem 109(4): 266–272. 17445872 10.1016/j.acthis.2007.02.007

[17] Sturm A, Leite AZ, Danese S, Krivacic KA, West GA, et al (2004) Divergent cell cycle kinetics underlie the distinct functional capacity of mucosal T cells in Crohn's disease and ulcerative colitis. Gut 53(11): 1624–1631. 15479683 10.1136/gut.2003.033613 PMC1774268

[18] Chau BN, Borges HL, Chen TT, Chen TT, Masselli A, et al (2002) Signal-dependent protection from apoptosis in mice expressing caspase-resistant Rb. Nat Cell Biol 4(10): 757–765. 12360286 10.1038/ncb853

[19] Borges HL, Bird J, Wasson K, Cardiff RD, Varki N, et al (2005) Tumor promotion by caspase-resistant retinoblastoma protein. Proc Natl Acad Sci U S A 102(43): 15587–15592. 16227443 10.1073/pnas.0503925102 PMC1255734

[20] Huang X, Masselli A, Frisch SM, Hunton IC, Jiang Y, et al (2007) Blockade of tumor necrosis factor-induced Bid cleavage by caspase-resistant Rb. J Biol Chem 282(40): 29401–29413. 17686781 10.1074/jbc.M702261200

[21] Pallone F, Blanco Gdel V, Vavassori P, Monteleone I, Fina D, et al (2003) Genetic and pathogenetic insights into inflammatory bowel disease. Curr Gastroenterol Rep 5: 487–492. 14602058 10.1007/s11894-003-0038-2

[22] Harvey RF, Bradshaw JM (1980) A simple index of Crohn's-disease activity. Lancet 1: 514. 6102236 10.1016/s0140-6736(80)92767-1

[23] Truelove SC, Witts LJ (1955) Cortisone in ulcerative colitis; final report on a therapeutic trial. Br Med J 4947: 1041–1048. 10.1136/bmj.2.4947.1041 PMC1981500 13260656

[24] Nirmalan NJ, Harnden P, Selby PJ, Banks RE (2009) Development and validation of a novel protein extraction methodology for quantitation of protein expression in formalin-fixed paraffin-embedded tissues using western blotting. J Pathol 217: 497–506. 19156775 10.1002/path.2504

[25] Bradford MM (1976) A rapid and sensitive method for the quantitation of microgram quantities of protein utilizing the principle of protein-dye binding. Anal Biochem 72: 248–254. 942051 10.1016/0003-2697(76)90527-3

[26] Dieckgraefe BK, Stenson WF, Korzenik JR, Swanson PE, Harrington CA (2000) Analysis of mucosal gene expression in inflammatory bowel disease by parallel oligonucleotide arrays. Physiol Genomics 4: 1–11. 11074008 10.1152/physiolgenomics.2000.4.1.1

[27] Uthoff SM, Eichenberger MR, Lewis RK, Fox MP, Hamilton CJ, et al (2001) Identification of candidate genes in ulcerative colitis and Crohn's disease using cDNA array technology. Int J Oncol 19: 803–810. 11562759 10.3892/ijo.19.4.803

[28] Costello CM, Mah N, Hasler R, Rosenstiel P, Waetzig GH (2005) Dissection of the inflammatory bowel disease transcriptome using genome-wide cDNA microarrays identifies novel candidate disease genes. PLoS Med 2: e199. 16107186 10.1371/journal.pmed.0020199 PMC1188246

[29] Knudson AG Jr (1971) Mutation and cancer: a statistical study of retinoblastoma. Proc Natl Acad Sci U S A 68: 820–923. 5279523 10.1073/pnas.68.4.820 PMC389051

[30] Harbour JW, Dean DC (2000) The Rb/E2F pathway: expanding roles and emerging paradigms. Genes Dev 14: 2393–2409. 11018009 10.1101/gad.813200

[31] Sherr CJ, McCormick F (2002) The RB and p53 pathways in cancer. Cancer Cell 2: 103–112. 12204530 10.1016/s1535-6108(02)00102-2

[32] Knudsen ES, Knudsen KE (2006) Retinoblastoma tumor suppressor: where cancer meets the cell cycle. Exp Biol Med (Maywood) 231: 1271–1281. 16816134 10.1177/153537020623100713

[33] Gotoh Y, Noda T, Iwakiri R, Fujimoto K, Rhoads CA, et al (2002) Lipid peroxide-induced redox imbalance differentially mediates CaCo-2 cell proliferation and growth arrest. Cell Prolif 35: 221–235. 12153614 10.1046/j.1365-2184.2002.00241.x PMC6496176

[34] Cicchillitti L, Fasanaro P, Biglioli P, Capogrosso MC, Martelli F (2003) Oxidative stress induces protein phosphatase 2A-dependent dephosphorylation of the pocket proteins pRb, p107, and p130. J Biol Chem 278: 19509–19517. 12621062 10.1074/jbc.M300511200

[35] Ying L, Marino J, Hussain SP, Khan MA, You S, et al (2005) Chronic inflammation promotes retinoblastoma protein hyperphosphorylation and E2F1 activation. Cancer Res 65(20): 9132–9136. 16230367 10.1158/0008-5472.CAN-05-1358

[36] Ying L, Hofseth AB, Browning DD, Nagarkatti M, Nagarkatti PS, et al (2007) Nitric oxide inactivates the retinoblastoma pathway in chronic inflammation. Cancer Res 67(19): 9286–9293. 17909036 10.1158/0008-5472.CAN-07-2238 PMC2752153

[37] Quinton JF, Sendid B, Reumaux D, Duthilleul P, Cortot A, et al (1998) Anti-*Saccharomyces cerevisiae* mannan antibodies combined with antineutrophil cytoplasmic autoantibodies in inflammatory bowel disease: prevalence and diagnostic role. Gut 42: 788–791. 9691915 10.1136/gut.42.6.788 PMC1727156

[38] Joossens S, Reinisch W, Vermeire S, Sendid B, Poulain D, et al (2002) The value of serologic markers in indeterminate colitis: a prospective follow-up study. Gastroenterology 122: 1242–1247. 11984510 10.1053/gast.2002.32980

[39] Israeli E, Grotto I, Gilburd B, Balicer RD, Goldin E, et al (2005) Anti-Saccharomyces cerevisiae and antineutrophil cytoplasmic antibodies as predictors of inflammatory bowel disease. Gut 54: 1232–1236. 16099791 10.1136/gut.2004.060228 PMC1774672

[40] Mokrowiecka A, Gasiorowska A, Malecka-Panas E (2007) pANCA and ASCA in the diagnosis of different subtypes of inflammatory bowel disease. Hepatogastroenterology 54(77): 1443–1448. 17708273

[41] Desplat-Jégo S, Johanet C, Escande A, Goetz J, Fabien N, et al (2007) Update on Anti-Saccharomyces cerevisiae antibodies, anti-nuclear associated anti-neutrophil antibodies and antibodies to exocrine pancreas detected by indirect immunofluorescence as biomarkers in chronic inflammatory bowel diseases: results of a multicenter study. World J Gastroenterol 13(16): 2312–8. 17511029 10.3748/wjg.v13.i16.2312 PMC4147139

[42] Torok HP, Glas J, Gruber R, Brumberger V, Strasser C, et al (2004) Inflammatory bowel disease-specific autoantibodies in HLA-B27-associated spondyloarthropathies: increased prevalence of ASCA and pANCA. Digestion 70: 49–54. 15308872 10.1159/000080081

[43] Krause I, Monselise Y, Milo G, Weinberger A (2002) Anti-*Saccharomyces cerevisiae* antibodies–a novel serologic marker for Behcet's disease. Clin Exp Rheumatol 20: S21–S24. 12371630

[44] Condino AA, Hoffenberg EJ, Accurso F, Penvari C, Anthony M, et al (2005) Frequency of ASCA seropositivity in children with cystic fibrosis. J Pediatr Gastroenterol Nutr 41: 23–26. 15990625 10.1097/01.mpg.0000166801.61708.60

[45] Kinzler KW, Vogelstein B (2002) Colorectal tumors. In: Vogelstein B, Kinzler KW. The Genetic Basis of Human Cancer, ed. McGraw-Hill, New York 583–612.

[46] Gavert N, Ben-Ze'ev A (2007) beta-Catenin signaling in biological control and cancer. J Cell Biochem 102(4): 820–828. 17854061 10.1002/jcb.21505

[47] Morin PJ, Sparks AB, Korinek V, Barker N, Clevers H, et al (1997) Activation of beta-catenin-Tcf signaling in colon cancer by mutations in beta-catenin or APC. Science 275: 1787–1790. 9065402 10.1126/science.275.5307.1787

[48] Iwamoto M, Ahnen DJ, Franklin WA, Maltzman TH (2000) Expression of β- catenin and full-length APC protein in normal and neoplastic colonic tissues. Carcinogenesis 21: 935–940. 10.1093/carcin/21.11.1935 11062151

[49] Han J, Soletti RC, Sadarangani A, Sridevi P, Ramirez ME, et al (2013) Nuclear expression of β-catenin promotes RB stability and resistance to TNF-induced apoptosis in colon cancer cells. Mol Cancer Res 11(3): 207–218. 23339186 10.1158/1541-7786.MCR-12-0670 PMC4191841

[50] Lee G, Goretsky T, Managlia E, Dirisina R, Singh AP, et al (2010) Phosphoinositide 3-kinase signaling mediates beta-catenin activation in intestinal epithelial stem and progenitor cells in colitis. Gastroenterology 139(3): 869–881. 20580720 10.1053/j.gastro.2010.05.037 PMC2930080

[51] Brown JB, Lee G, Managlia E, Grimm GR, Dirisina R, et al (2010) Mesalamine inhibits epithelial beta-catenin activation in chronic ulcerative colitis. Gastroenterology 138(2): 595–605. 19879273 10.1053/j.gastro.2009.10.038 PMC2819654

